# Negative Effects of Chronic Rapamycin Treatment on Behavior in a Mouse Model of Fragile X Syndrome

**DOI:** 10.3389/fnmol.2017.00452

**Published:** 2018-01-12

**Authors:** Rachel M. Saré, Alex Song, Inna Loutaev, Anna Cook, Isabella Maita, Abigail Lemons, Carrie Sheeler, Carolyn B. Smith

**Affiliations:** Section on Neuroadaptation and Protein Metabolism, National Institute of Mental Health, National Institutes of Health, United States Department of Health and Human Services, Bethesda, MD, United States

**Keywords:** Fragile X, rapamycin, mTORC1, hyperactivity, anxiety, social behavior, learning and memory, sleep

## Abstract

Fragile X syndrome (FXS), the most common form of inherited intellectual disability, is also highly associated with autism spectrum disorders (ASD). It is caused by expansion of a CGG repeat sequence on the X chromosome resulting in silencing of the *FMR1* gene. This is modeled in the mouse by deletion of *Fmr1* (*Fmr1* KO). *Fmr1* KO mice recapitulate many of the behavioral features of the disorder including seizure susceptibility, hyperactivity, impaired social behavior, sleep problems, and learning and memory deficits. The mammalian target of rapamycin pathway (mTORC1) is upregulated in *Fmr1* KO mice and is thought to be important for the pathogenesis of this disorder. We treated *Fmr1* KO mice chronically with an mTORC1 inhibitor, rapamycin, to determine if rapamycin treatment could reverse behavioral phenotypes. We performed open field, zero maze, social behavior, sleep, passive avoidance, and audiogenic seizure testing. We found that pS6 was upregulated in *Fmr1* KO mice and normalized by rapamycin treatment, but, except for an anxiogenic effect, it did not reverse any of the behavioral phenotypes examined. In fact, rapamycin treatment had an adverse effect on sleep and social behavior in both control and *Fmr1* KO mice. These results suggest that targeting the mTOR pathway in FXS is not a good treatment strategy and that other pathways should be considered.

## Introduction

Fragile X syndrome is the most common inherited form of intellectual disability. It is an X-linked disorder caused by an elongation of a CGG repeat (>200) in the 5′-untranslated region of the *FMR1* gene leading to silencing of the gene and a paucity of the protein product FMRP (Verkerk et al., 1991). FXS primarily affects males who experience a myriad of symptoms ranging from cognitive impairment, seizures, disordered sleep, and emotional instability (Loesch et al., 2002; Jin and Warren, 2003; Penagarikano et al., 2007; Kronk et al., 2010). Up to 60% of FXS cases are diagnosed with ASD (Hagerman et al., 1986; Bailey et al., 1998). In a mouse model of FXS, *Fmr1* KO, behavioral characteristics paralleling those seen in FXS individuals have been demonstrated. These include hyperactivity, decreased preference for social novelty, sleep deficits, and learning and memory deficits (Mineur et al., 2002; Qin et al., 2002; Kooy, 2003; Spencer et al., 2005; Liu et al., 2011; Kazdoba et al., 2014; Saré et al., 2016, 2017).

It is thought that a dysregulation of protein synthesis, particularly at synapses, underlies these behavioral symptoms. Two signaling pathways that are critical nodes in the regulation of protein synthesis may be involved: mitogen activated protein kinase/extracellular signal-regulated kinase (MAPK/ERK) and mTOR. mTOR in association with raptor forms mTOR complex 1 (mTORC1), a metabolic sensor and a key regulator of cell growth. Dysregulation of mTORC1 is associated with many neurological disorders including ASD, epilepsy, and neurodegenerative disorders (Lipton and Sahin, 2014). The MAPK/ERK cascade functions in cell proliferation, differentiation, and survival, and is activated through growth factors acting on receptor-activated tyrosine kinases. Both pathways have been shown to be elevated in studies of *Fmr1* KO mice (Osterweil et al., 2010; Sharma et al., 2010; Bhattacharya et al., 2012; Liu et al., 2012; Busquets-Garcia et al., 2013; Choi et al., 2016; Sawicka et al., 2016). However, there is some debate whether the mTORC1 pathway is actually elevated in *Fmr1* KO mice since some studies have not found increases in pathway components (Osterweil et al., 2010; Sawicka et al., 2016).

In the present study, we sought to determine the effects of rapamycin-induced mTORC1 inhibition on the behavioral phenotype of *Fmr1* KO mice. We chronically treated control and *Fmr1* KO mice by dietary administration of rapamycin. We found that p-S6 (a downstream target of both mTORC1 and ERK) in cortical lysates from vehicle-treated *Fmr1* KO mice was elevated compared to vehicle-treated controls. Levels of p-S6 were reduced in *Fmr1* KO mice following rapamycin treatment. We performed a battery of behavioral tests to examine sleep duration, activity, anxiety-like behavior, social behavior, learning and memory, and seizure susceptibility. Paradoxically, we found that rapamycin did not reverse most of the phenotypes examined. In fact, in both control and *Fmr1* KO mice, rapamycin decreased sleep duration and measures of social interaction. Our results suggest that the mTORC1 pathway is not causally involved in the behavioral phenotype of FXS and that alternate pathways should be considered for targeted treatments.

## Materials and Methods

### Animals

Male *Fmr1* KO and control animals on a C57BL/6J background were generated from heterozygous and control breeder pairs in-house. Animals were weaned at 21 days of age. Genotyping was performed from tail biopsies as previously described (Qin et al., 2002). Animals were group housed in a climate controlled facility with access to food and water *ad libitum*. All procedures were performed in accordance with the National Institutes of Health Guidelines on the Care and Use of Animals and approved by the National Institute of Mental Health Animal Care and Use Committee.

### Rapamycin Treatment

At 21 days of age (P21), males in each litter were separated such that half received vehicle treatment and the other half received rapamycin treatment. Rapamycin was encapsulated with Eudragit (Rapamycin Holdings, San Antonio, TX, United States) and incorporated into mouse chow at 11 mg/kg food (Purina LabDiet, St. Louis, MO, United States). Based on how much food an average mouse consumes per day (4g; Tremblay et al., 2007), P21 animals received 4.4 mg/kg/day. Once the animals reached adult weight around 60 days of age, we estimate that the animals received 1.75 mg/kg/day. This dosage was based on a previous dosage used to reverse social behavior deficits in a mouse model of TSC (Reith et al., 2012).

### Behavior Testing

Beginning at 60 days of age, animals were subjected to a battery of behavior tests with tests spaced 1 week apart. They were conducted from the least stressful to the most stressful in the following order: sleep testing, open field, zero maze, social behavior, and passive avoidance.

### Sleep Monitoring

Sleep testing was conducted by means of a home-cage monitoring system (Columbus Instruments, Columbus, OH, United States) as previously described (Saré et al., 2017). Briefly, animals were individually housed and recorded for 72 consecutive hours. To eliminate the effect of habituation to the home-cage environment and single housing (Saré et al., 2017), we analyzed sleep duration from the last 48 h of recording. An animal was considered asleep if it had 40 s of consecutive inactivity. The percent time asleep was calculated separately for the light (inactive) and the dark (active) phases.

### Open Field

Activity and anxiety were assayed by means of photobeam detection in a novel open field environment (Coulbourn Instruments, Whitehall, PA, United States) as previously described (Saré et al., 2015). Briefly, total horizontal distance moved was recorded over a 30-min session in 5 min epochs as a measure of total activity. The ratio of horizontal distance traveled in the center (more than 1.91 cm away from the arena walls) to total distance was calculated for each epoch as an inverse measure of anxiety.

### Zero Maze

Anxiety was assayed by means of a zero maze (Med Associates, Fairfax, VT, United States). Animals were placed facing the open portion of the zero maze and allowed to explore the maze for 5 min. The times spent in the open and closed portions of the maze were determined. An animal was considered to be in a portion of the maze if both forepaws crossed into that portion. If an animal fell off the maze, it was eliminated from the analysis.

### Social Behavior

Social behavior was assayed by means of a 3-chambered apparatus as previously described (Saré et al., 2016). The assay had three consecutive parts. First, the animal was allowed a 5 min habituation period to the empty chamber. Animals demonstrating a side preference of more than 3 min were eliminated from the test. Second, in the sociability phase, an age-matched stranger mouse was placed in a sociability enclosure (Noldus Information Technology, Inc., Leesburg, VA, United States) in one chamber. An empty sociability enclosure was placed in the opposite chamber. These locations were alternated between mice to avoid a side bias. The test animal was placed in the center chamber and allowed to freely explore all chambers for 5 min. Times spent sniffing the sociability enclosures were recorded by subsequent video analysis. Sniffing was analyzed by means of the TopScan software (Clever Systems, Reston, VA, United States). Parameters were set to define sniffing as the animal’s nose directed toward the enclosure and within 20 mm of the enclosure. Third, in the social novelty phase, a novel stranger mouse was added to the previously empty sociability enclosure. The test animal was once again allowed to explore for 5 min, with measures recorded as before.

### Passive Avoidance

Passive avoidance was conducted as previously reported (Saré et al., 2016). Briefly, habituation was conducted on Day 1. The animals were placed in the lighted chamber of the passive avoidance shuttle box with the door to the dark chamber closed. After 30 s, the door to the dark chamber opened. Once the animal entered, the test was ended. On Day 2, the animal was subjected to two training sessions. The animal was once again placed in the lighted chamber with the door to the dark chamber closed. After 30 s, the door was opened. Once the animal entered the dark chamber, it received a footshock (0.3 mA, 1 s). The animal was left in the dark chamber for 15 s and then moved to a holding cage for 120 s and was then placed in the lighted chamber and the process was repeated. On Day 3 and 24 h after the training session, animals were placed in the lighted chamber with the door to the dark chamber closed. The door was opened and the latency to enter the dark chamber was recorded with a cutoff of 570 s.

### Audiogenic Seizure Susceptibility

In separate groups of animals, we conducted audiogenic seizure testing as previously reported (Miano et al., 2008). Testing began at P30. Animals were placed in a sound attenuating chamber with a viewing window (Med Associates, Fairfax, VT, United States). A siren (130 dB) sounded (Wal-Mart, Bentonville, AR, United States) for 5 min while seizure activity was observed. A seizure was defined as wild running, sometimes followed by myoclonic convulsions, sometimes followed by respiratory arrest. The frequency of each of these behaviors occurring in response to the tone was recorded.

### Western Blotting

Mice used for Western blotting had been tested for behavior with the exception of passive avoidance and audiogenic seizure susceptibility. A week after the last behavioral test, mice were decapitated and the frontal cortex was rapidly dissected for total protein extraction. The tissue was weighed and homogenized with 5% (weight/volume) solution of Tissue Protein Extraction Reagent (Thermo Fisher Scientific, Waltham, MA, United States) with 1% Halt Protease and Phosphatase Inhibitor Cocktail (Thermo Fisher Scientific) and 1% EDTA (Thermo Fisher Scientific). The homogenate was centrifuged at 15,000 × *g* for 15 min at 4°C and the supernatant was collected. Protein extracts (10 μg) were treated with equal volume 2× Laemmli buffer, incubated at 70°C for 10 min, and run on a 4–15% Mini-PROTEAN TGX Stain-Free gel (Bio-Rad Laboratories, Hercules, CA, United States). The gel was activated under ultraviolet light, proteins transferred to a nitrocellulose membrane, incubated with primary antibody overnight at 4°C, followed by secondary antibody [goat anti-rabbit horseradish peroxidase-linked at 1:10,000 (Bio-Rad Laboratories)] for 1 h at room temperature. We employed the Stain-Free Technology (Bio-Rad Laboratories) to normalize blots to total protein. The membrane was imaged under Stain-Free to determine total protein for loading control. The membrane was then incubated in Clarity substrate and imaged by means of a chemiluminescent signal on a ChemiDoc MP Imager (Bio-Rad Laboratories). Primary antibodies were used at a 1:1000 dilution and were as follows: FMRP (Abcam 27455), p-mTOR (Cell Signaling 5536), mTOR (Cell Signaling 2983), p-p70S6K (Cell Signaling 9234), p70S6K (Cell Signaling 2708), p-S6 235/236 (Cell Signaling 2211), p-S6 240/244 (Cell Signaling 2215), S6 (Cell Signaling 2217), p-ERK (Cell Signaling 4370), ERK (Cell Signaling 7124), p-AKT Ser473 (Cell Signaling 4060), and AKT (Cell Signaling 9272). Values presented are relative to the mean of vehicle-treated controls.

### Statistical Analysis

Data are reported as means ± SEM. Data from the zero maze, passive avoidance tests, and Westerns blots were analyzed by means of two-way ANOVA with genotype (control, *Fmr1* KO) and condition (vehicle, rapamycin) as between subjects variables. Sleep and open field data were analyzed by means of a mixed-model repeated measures three-way ANOVA with genotype (control, *Fmr1* KO) and condition (vehicle, rapamycin) as between subjects variables and phase (sleep) or epoch (open field) as within subjects variables. In cases in which the interaction between or among variables was statistically significant, we compared groups and/or conditions by means of *post hoc t*-tests. Social behavior was analyzed by means of paired student’s *t*-tests to compare stranger vs. object (sociability) or familiar vs. novel (social novelty). Audiogenic seizures were analyzed by means of a Fisher’s exact test comparing the effect of treatment in *Fmr1* KO animals. Effects of *p* ≤ 0.05 are considered statistically significant and are denoted with a “^∗^.” Effects of 0.10 ≥*p* > 0.05 are also reported here and are denoted with a “∼.” A table reporting the *F*-values and corresponding *p*-values for interactions and main effects are listed for the Western blots (**Table 1**) and all behavior tests analyzed with ANOVA (**Table 2**).

**Table 1:** ANOVA results for Western blots.

Protein	Interaction	Main effect	*F*_(df,error)_ value	*P*-value
p-mTOR	Genotype × treatment		*F*_(1,10)_= 0.593	0.459
		Genotype	*F*_(1,10)_= 0.002	0.964
		Treatment	*F*_(1,10)_= 0.283	0.606
mTOR	Genotype × treatment		*F*_(1,10)_= 0.433	0.526
		Genotype	*F*_(1,10)_= 0.735	0.411
		Treatment	*F*_(1,10)_= 0.060	0.811
p-mTOR/mTOR	Genotype × treatment		*F*_(1,10)_= 0.003	0.956
		Genotype	*F*_(1,10)_= 0.534	0.482
		Treatment	*F*_(1,10)_= 0.084	0.778
p-p70S6k	Genotype × treatment		*F*_(1,10)_ = 0.059	0.813
		Genotype	*F*_(1,10)_ = 0.029	0.868
		Treatment	*F*_(1,10)_ = 0.090	0.770
p70S6k	Genotype × treatment		*F*_(1,10)_ = 0.148	0.709
		Genotype	*F*_(1,10)_ = 1.912	0.197
		Treatment	*F*_(1,10)_ = 0.785	0.396
p-p70S6k/p70S6k	Genotype × treatment		*F*_(1,10)_ = 0.096	0.763
		Genotype	*F*_(1,10)_ = 0.351	0.567
		Treatment	*F*_(1,10)_ = 0.007	0.936
p-S6 235/236	Genotype × treatment		*F*_(1,10)_= 11.177	0.007^∗∗^
		Genotype	*F*_(1,10)_= 6.098	0.033^∗^
		Treatment	*F*_(1,10)_= 8.667	0.015^∗^
p-S6 240/244	Genotype × treatment		*F*_(1,10)_= 7.258	0.023^∗^
		Genotype	*F*_(1,10)_= 3.273	0.101
		Treatment	*F*_(1,10)_= 6.800	0.026^∗^
S6	Genotype × treatment		*F*_(1,10)_= 0.446	0.520
		Genotype	*F*_(1,10)_= 1.949	0.193
		Treatment	*F*_(1,10)_= 2.55	0.141
p-S6 (235/236)/S6	Genotype × treatment		*F*_(1,10)_= 4.843	0.052^∼^
		Genotype	*F*_(1,10)_= 9.880	0.011^∗^
		Treatment	*F*_(1,10)_= 0.004	0.948
p-S6 (240/244)/S6	Genotype × treatment		*F*_(1,10)_= 4.138	0.069^∼^
		Genotype	*F*_(1,10)_= 4.226	0.067
		Treatment	*F*_(1,10)_= 0.659	0.436
p-AKT 473	Genotype × treatment		*F*_(1,10)_= 6.601	0.028^∗^
		Genotype	*F*_(1,10)_= 1.840	0.205
		Treatment	*F*_(1,10)_= 3.844	0.078^∼^
AKT	Genotype × treatment		*F*_(1,10)_= 0.322	0.583
		Genotype	*F*_(1,10)_= 0.215	0.653
		Treatment	*F*_(1,10)_= 0.069	0.797
p-AKT (473)/Akt	Genotype × treatment		*F*_(1,10)_= 1.705	0.221
		Genotype	*F*_(1,10)_= 0.346	0.570
		Treatment	*F*_(1,10)_= 3.008	0.114
p-ERK	Genotype × treatment		*F*_(1,10)_= 1.489	0.250
		Genotype	*F*_(1,10)_= 0.122	0.734
		Treatment	*F*_(1,10)_= 6.925	0.025^∗^
ERK	Genotype × treatment		*F*_(1,10)_= 0.000	0.986
		Genotype	*F*_(1,10)_= 0.477	0.505
		Treatment	*F*_(1,10)_= 5.517	0.041^∗^
p-ERK/ERK	Genotype × treatment		*F*_(1,10)_= 1.001	0.341
		Genotype	*F*_(1,10)_= 0.013	0.912
		Treatment	*F*_(1,10)_= 1.151	0.309

*∼0.05 ≤*p* ≤ 0.1; ^∗^0.01 ≤*p* ≤ 0.05; ^∗∗^0.001 ≤*p* ≤ 0.01.*

**Table 2:** Repeated measures ANOVA results for behavior testing.

Behavior	Interaction	Main effect	*F*_(df,error)_ value	*P*-value
Open field				
Horizontal distance moved	Genotype × treatment × epoch		*F*_(4,366)_= 0.255	0.919
	Treatment × epoch		*F*_(4,366)_= 2.292	0.054∼
	Genotype × epoch		*F*_(4,366)_= 0.559	0.707
	Genotype × treatment		*F*_(1,84)_= 0.270	0.605
		Genotype	*F*_(1,84)_= 8.154	0.005*
		Treatment	*F*_(1,84)_= 1.536	0.219
		Epoch	*F*_(4,366)_= 170.930	<0.001*
Center/total distance	Genotype × treatment × epoch		*F*_(4,360)_= 0.899	0.470
	Treatment × epoch		*F*_(4,360)_= 0.643	0.643
	Genotype × epoch		*F*_(4,360)_= 0.823	0.518
	Genotype × treatment		*F*_(1,85)_= 0.136	0.713
		Genotype	*F*_(1,85)_= 3.081	0.083∼
		Treatment	*F*_(1,85)_= 3.115	0.081∼
		Epoch	*F*_(4,360)_= 7.724	<0.001
Zero maze	Genotype × treatment		*F*_(1,87)_= 0.250	0.619
		Genotype	*F*_(1,87)_= 13.059	0.001*
		Treatment	*F*_(1,87)_= 5.111	0.026*
Passive avoidance	Genotype × treatment		*F*_(1,74)_= 0.045	0.832
		Genotype	*F*_(1,74)_= 5.004	0.028*
		Treatment	*F*_(1,74)_= 0.027	0.871
Sleep				
Total sleep time	Genotype × treatment × phase		*F*_(1,102)_= 0.020	0.887
	Treatment × phase		*F*_(1,102)_= 0.236	0.628
	Genotype × phase		*F*_(1,102)_= 3.558	0.062∼
	Genotype × treatment		*F*_(1,102)_= 0.374	0.542
		Genotype	*F*_(1,102)_= 5.324	0.023*
		Treatment	*F*_(1,102)_= 4.056	0.047*
		Phase	*F*_(1,102)_= 1494.025	<0.001*

*∼0.05 < *p* ≤ 0.1; ^∗^*p* ≤ 0.05.*

## Results

### Increased mTORC1 Activity in *Fmr1* KO Mice Normalized by Chronic Rapamycin Treatment

**FIGURE 1:**
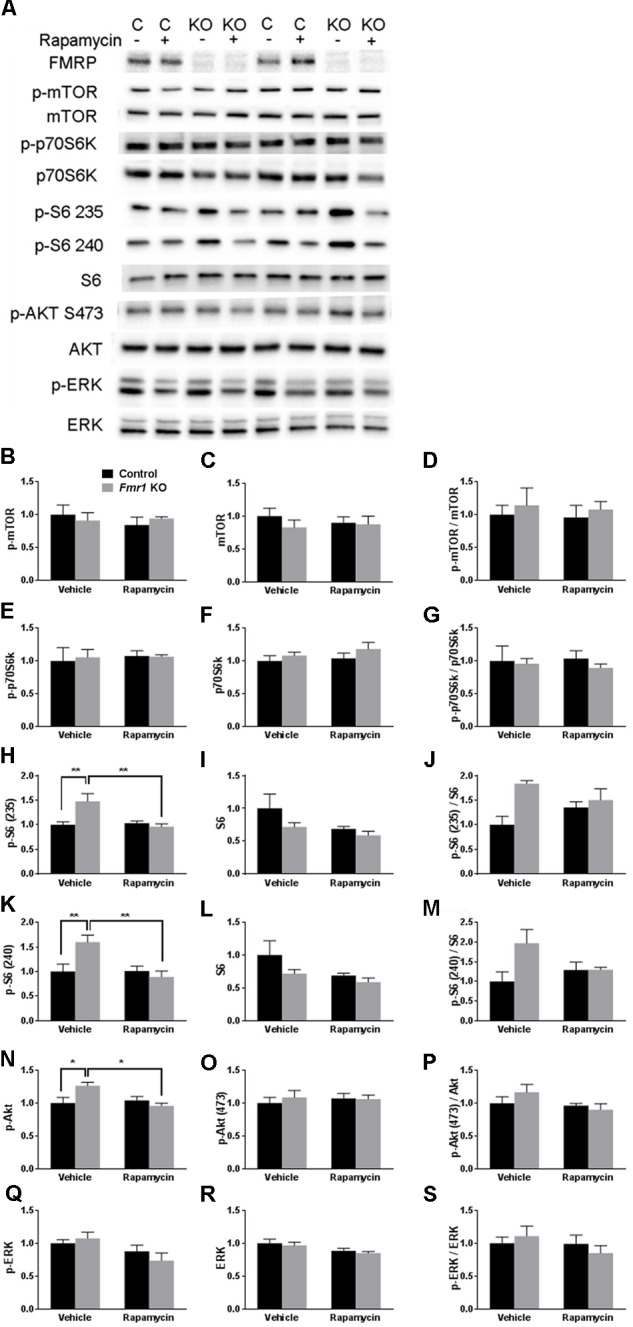
Westerns blots of frontal cortex of *Fmr1* KO and control mice on vehicle and rapamycin treatment. **(A)** Representative Western blot images. **(B)** p-mTOR levels did not differ among the groups. **(C)** mTOR levels did not differ among the groups. **(D)** p-mTOR/Total mTOR did not differ among the groups. **(E)** p-p70S6k did not differ among the groups. **(F)** p70S6k did not differ among the groups. **(G)** p-p70S6k/Total p70S6k did not differ among the groups. **(H)** The genotype × treatment interaction for pS6 235/236 was statistically significant. *Post hoc t*-tests revealed that vehicle-treated *Fmr1* KO animals had significantly higher p-S6 235/236 (*p* = 0.002) compared to vehicle-treated controls. This was significantly reduced by rapamycin treatment (*p* = 0.002). **(I)** S6 levels did not differ among groups. **(J)** The genotype × treatment interaction for p-S6 (235/236)/Total S6 approached statistical significance. We looked at individual differences by means of *post hoc t*-tests and found that the difference between vehicle-treated controls and vehicle-treated *Fmr1* KO mice was statistically significant (*p* = 0.004). **(K)** The genotype × treatment interaction for p-S6 240/244 was statistically significant. (**Post hoc t*-tests revealed that vehicle-treated *Fmr1* KO animals had significantly higher p-S6 240/244 levels compared to vehicle-treated controls (*p* = 0.010). This was significantly reduced with rapamycin treatment (*p* = 0.006). **(L)** Total S6 levels did not differ among the groups. **(M)** The genotype × treatment interaction for p-S6 (240/244)/Total S6 approached statistical significance. We looked at individual differences by means of *post hoc t*-tests and found that the difference between vehicle-treated controls and vehicle-treated *Fmr1* KO mice was statistically significant (*p* = 0.016). **(N)** The genotype × treatment interaction for p-AKT Ser473 was statistically significant. *Post hoc t*-tests revealed that vehicle-treated *Fmr1* KO animals had higher p-AKT compared to vehicle-treated controls (*p* = 0.020). p-AKT Ser473 levels were reduced in *Fmr1* KO animals after rapamycin treatment (*p* = 0.013). **(O)** Total AKT levels did not differ among the groups. **(P)** p-Akt (473)/Akt did not differ among the groups. **(Q)** The main effect of treatment for p-ERK levels was statistically significant indicating that regardless of genotype, rapamycin reduced p-ERK. **(R)** The main effect of treatment for ERK levels was statistically significant indicating that regardless of genotype, rapamycin reduced ERK. **(S)** p-ERK/ERK did not differ among the groups. **(B–S)** Levels were normalized to total protein in the blot. Values presented are relative to the mean of vehicle-treated control values. Bars represent mean ± SEM. ∼0.05 < *p* ≤ 0.1, ^∗^0.01 ≤ *p* ≤ 0.05, ^∗∗^0.001 ≤ *p* ≤ 0.01 as determined by *post hoc t*-tests. *n* = 4 vehicle-treated control, *n* = 4 rapamycin-treated control, *n* = 3 vehicle-treated *Fmr1* KO, *n* = 3 rapamycin-treated *Fmr1* KO.*)

We analyzed lysates of frontal cortex from vehicle- and rapamycin-treated *Fmr1* KO and control mice. FMRP expression was absent in all *Fmr1* KO mice regardless of treatment (**Figure 1A**). In **Figure 1**, we present the results of the effects of genotype and rapamycin treatment on the phosphorylated forms (**Figures 1B,E,H,K,N,Q**), total (**Figures 1C,F,I,L,O,R**) and the ratio of phosphorylated to total (**Figures 1D,G,J,M,P,S**) for the signaling molecules measured. We did not find either an effect of genotype or of treatment on p-mTOR (**Figure 1B** and **Table 1**). We also did not find either an effect of genotype or treatment on p-p70S6k (**Figure 1E** and **Table 1**). We examined two phosphorylation sites on S6 (235/236 and 240/244), and in both cases, the genotype × treatment interactions were statistically significant (**Table 1**). *Post hoc t*-tests indicate that vehicle-treated *Fmr1* KO mice had significantly higher p-S6 than vehicle-treated controls (*p* = 0.010 for 235/236) (*p* = 0.041 for 240/244). For both sites, rapamycin reduced p-S6 levels in *Fmr1* KO mice (*p* = 0.009 for 235/236) (*p* = 0.024 for 240/244), but had no effect on controls (**Figures 1A,H,K**). To examine the mTORC2 pathway, we also found a statistically significant genotype × treatment interaction for p-AKT Ser473 (**Table 1**). *Post hoc t*-tests indicate that vehicle-treated *Fmr1* KO animals had increased p-AKT relative to vehicle-treated controls (*p* = 0.02). Rapamycin reduced p-AKT levels in *Fmr1* KO mice (*p* = 0.013), but had no effect on controls (**Figures 1A,N**). We also examined p-ERK. Although there was no genotype × treatment interaction, we did detect a statistically significant main effect of treatment for both p-ERK and total ERK. Regardless of genotype, rapamycin treatment decreased p-ERK and ERK levels (**Figures 1A,Q,R** and **Table 1**).

Given that p-S6 activity was higher in *Fmr1* KO mice and reduced by rapamycin treatment, we hypothesized that rapamycin treatment would ameliorate the behavioral phenotypes in *Fmr1* KO mice.

### Rapamycin Did Not Alter Hyperactivity in *Fmr1* KO Mice

**FIGURE 2:**
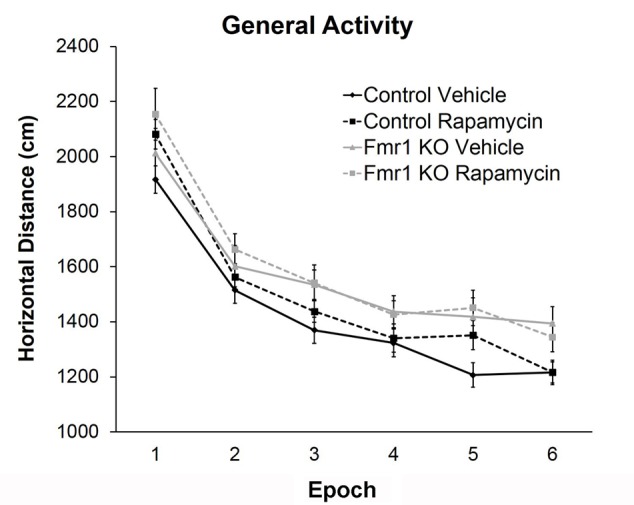
Rapamycin did not reverse hyperactivity in *Fmr1* KO mice. Based on the total distance moved in an open field, there was a main effect of genotype (*p* = 0.005), indicating that regardless of treatment, *Fmr1* KO mice were hyperactive compared to controls. There was also a near significant treatment × epoch interaction (*p* = 0.054) indicating a potential change in habituation. Points represent mean ± SEM in 26 vehicle-treated control, 23 rapamycin-treated control, 8 vehicle-treated *Fmr1* KO, and 21 rapamycin-treated *Fmr1* KO.

To examine hyperactivity, we conducted open field testing. We found a statistically significant main effect of genotype regardless of treatment, indicating that *Fmr1* KO mice were hyperactive compared to controls, as previously reported (Liu et al., 2011; **Figure 2** and **Table 2**). There was also a near statistically significant treatment × epoch interaction (*p* = 0.054) suggesting that rapamycin may enhance reactivity to the novel environment, regardless of genotype.

### Rapamycin Increased Anxiety-Like Behavior in *Fmr1* KO and Control Mice

**FIGURE 3:**
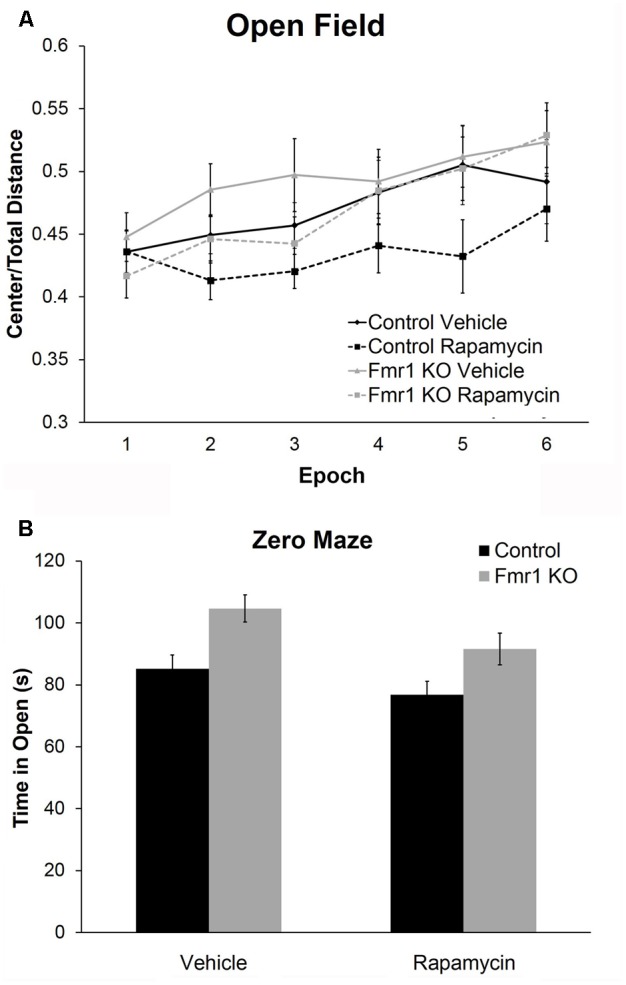
Rapamycin increased anxiety in control and *Fmr1* KO mice. **(A)** Regarding the ratio of distance traveled in the center to total distance traveled in the open field, there was a near significant main effect of genotype (*p* = 0.083) suggesting that *Fmr1* KO mice tended to show a decrease in anxiety. There was also a near significant main effect of treatment (*p* = 0.081) suggesting that, regardless of genotype, rapamycin may have increased anxiety. Points represent mean ± SEM in 26 vehicle-treated control, 23 rapamycin-treated control, 18 vehicle-treated *Fmr1* KO, and 21 rapamycin-treated *Fmr1* KO. **(B)** In the zero maze, there was a significant main effect of genotype (*p* = 0.001) showing that *Fmr1* KO mice spent more time in the open arms compared to control mice. Again, this shows that *Fmr1* KO mice have reduced anxiety. There was also a significant main effect of treatment (*p* = 0.026) showing that, regardless of genotype, rapamycin decreased the time spent in the open arms, indicating that rapamycin increases anxiety. Bars represent mean ± SEM in 27 vehicle-treated control, 28 rapamycin-treated control, 16 vehicle-treated *Fmr1* KO, and 19 rapamycin-treated *Fmr1* KO.

To examine anxiety, we analyzed the ratio of distance traveled in the center of the open field to total distance traveled. The main effect of epoch was statistically significant (*p* < 0.001) indicating that regardless of genotype or treatment, mice became less anxious as the test progressed (**Figure 3A** and **Table 2**). Main effects of genotype and treatment approached statistical significance suggesting that anxiety may be lower in *Fmr1* KO mice regardless of treatment and that in both genotypes, rapamycin treatment tended to increase anxiety-like behavior.

As another measure of anxiety-like behavior, we tested animals in the zero maze (**Figure 3B**). In this test, the main effects of both genotype and treatment were statistically significant (**Table 2**). Zero maze results are in accord with open field behavior. Our results show that regardless of treatment, *Fmr1* KO mice spent more time in the open arms indicating that *Fmr1* KO mice are less anxious compared to controls as previously reported (Liu et al., 2011). Moreover, regardless of genotype, rapamycin decreased the time spent in the open arms indicating that rapamycin increased anxiety-like behavior in both genotypes.

### Rapamycin Did Not Improve Performance on the Passive Avoidance Test in *Fmr1* KO Mice

**FIGURE 4:**
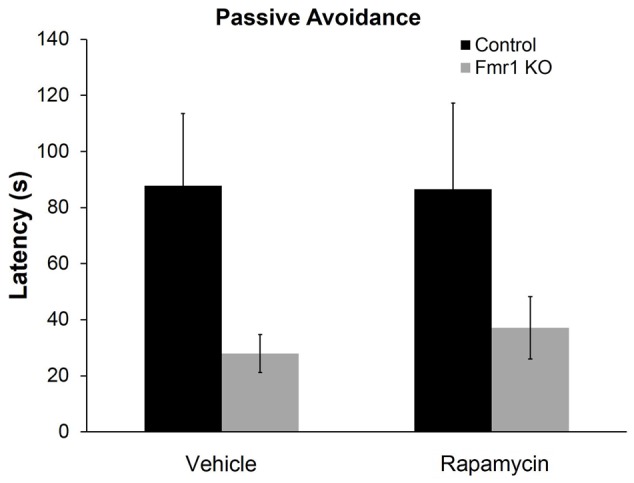
Rapamycin did not reverse deficits on the passive avoidance test in *Fmr1* KO mice. In the passive avoidance test, there was a statistically significant main effect of genotype (*p* = 0.028) indicating that, regardless of treatment, *Fmr1* KO mice have a significantly reduced latency to enter the dark side of the passive avoidance apparatus. This suggests impaired learning and memory. This deficit was not affected by rapamycin treatment. Bars represent mean ± SEM for 22 vehicle-treated control, 23 rapamycin-treated control, 15 vehicle-treated *Fmr1* KO, and 18 rapamycin-treated *Fmr1* KO.

To examine a form of fear learning, we tested mice on passive avoidance (**Figure 4**). We found a statistically significant main effect of genotype (*p* = 0.028) (**Table 2**), indicating that regardless of treatment, *Fmr1* KO mice had a lower latency to enter the dark side. The main effect of rapamycin treatment was not statistically significant indicating that rapamycin did not enhance performance on this test.

### Rapamycin Decreased Sleep Duration in *Fmr1* KO and Control Mice

**FIGURE 5:**
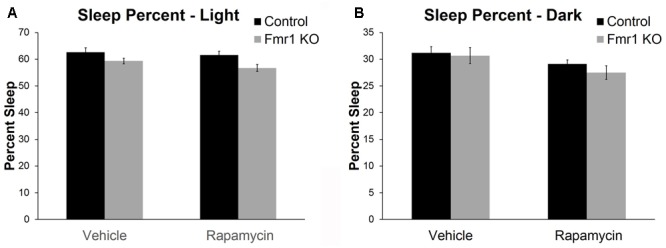
Rapamycin resulted in reduced sleep duration in control and *Fmr1* KO mice. **(A,B)** Activity-based monitoring for sleep detection indicates a near significant genotype × phase interaction (*p* = 0.062). This suggests that, regardless of treatment, *Fmr1* KO mice had reduced sleep duration in the light phase compared to controls. We also found a significant main effect of treatment (*p* = 0.047) showing that, regardless of phase or genotype, rapamycin resulted in reduced sleep duration. Bars represent mean ± SEM for 32 vehicle-treated control, 34 rapamycin-treated control, 20 vehicle-treated *Fmr1* KO, and 20 rapamycin-treated *Fmr1* KO.

Sleep in the active and inactive phases was assessed by home-cage monitoring. We found a nearly statistically significant genotype × phase interaction (*p* = 0.062) (**Table 2**). This is consistent with previous work indicating that decreases in sleep duration occurred primarily in the inactive phase in *Fmr1* KO (Saré et al., 2017). We also found a statistically significant main effect of treatment (*p* = 0.047) (**Table 2**). These results indicate that rapamycin treatment reduced sleep duration across genotypes and phases (**Figure 5**).

### Rapamycin Impaired Social Behavior in *Fmr1* KO and Control Mice

**FIGURE 6:**
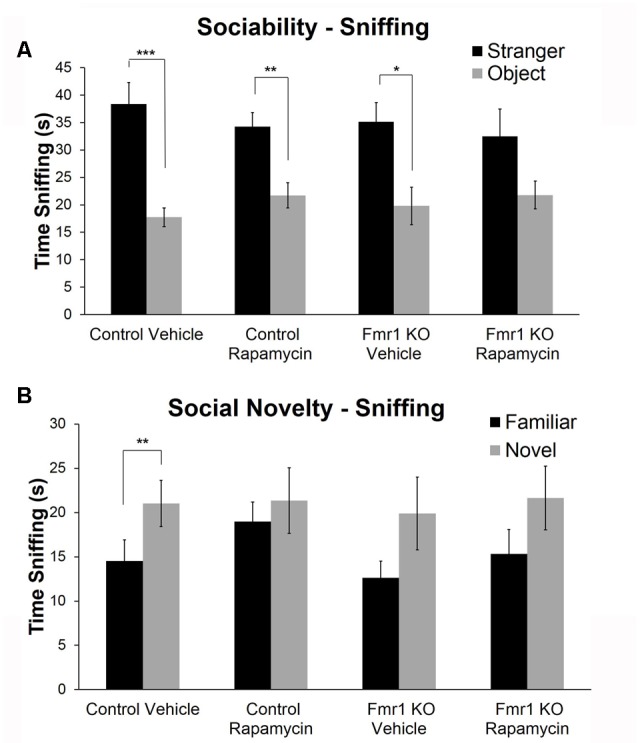
Rapamycin adversely affected social behavior in both *Fmr1* KO and control mice. **(A)** For sociability, control mice on both vehicle and rapamycin treatment as well as *Fmr1* KO mice on vehicle treatment showed a significant preference for interacting with the mouse compared to the object. *Fmr1* KO mice on rapamycin treatment did not show a preference. This suggests that rapamycin treatment induced a sociability deficit in *Fmr1* KO mice. Bars are means ± SEM in 26 vehicle-treated control, 28 rapamycin-treated control, 17 vehicle-treated *Fmr1* KO, and 20 rapamycin-treated *Fmr1* KO. **(B)** For the preference for social novelty, only control animals on vehicle treatment showed a preference for the novel mouse. Control mice on rapamycin treatment did not show a preference indicating that rapamycin induced a social behavior abnormality in control animals. Neither *Fmr1* KO animals on vehicle or rapamycin showed a preference for the novel mouse indicating that rapamycin did not rescue the preference for social novelty in *Fmr1* KO mice. Bars are means ± SEM in 26 vehicle-treated control, 28 rapamycin-treated control, 17 vehicle-treated *Fmr1* KO, and 17 rapamycin-treated *Fmr1* KO. ^∗^*p* < 0.05, ^∗∗^*p* < 0.01, ^∗∗∗^*p* < 0.001 as determined by paired *t*-tests.

To examine social behavior, we used the three-chambered apparatus to assess sociability and preference for social novelty. In the test of sociability, vehicle (*p* = 0.0003)- and rapamycin (*p* = 0.005)-treated control mice and vehicle-treated *Fmr1* KO mice (*p* = 0.02) had a significant preference for the mouse compared to the object, but *Fmr1* KO mice on rapamycin treatment did not (**Figure 6A**). This indicates that rapamycin impaired sociability in *Fmr1* KO mice.

In the test of preference for social novelty, only the control mice on vehicle treatment had a significant preference for the novel mouse (*p* = 0.003) (**Figure 6B**). This was abolished with rapamycin treatment. Additionally and as previously reported (Liu et al., 2011), *Fmr1* KO animals did not show a preference for the novel mouse. This was not changed with rapamycin treatment (**Figure 6B**).

### Rapamycin Did Not Reverse Audiogenic Seizure Susceptibility in *Fmr1* KO Mice

**FIGURE 7:**
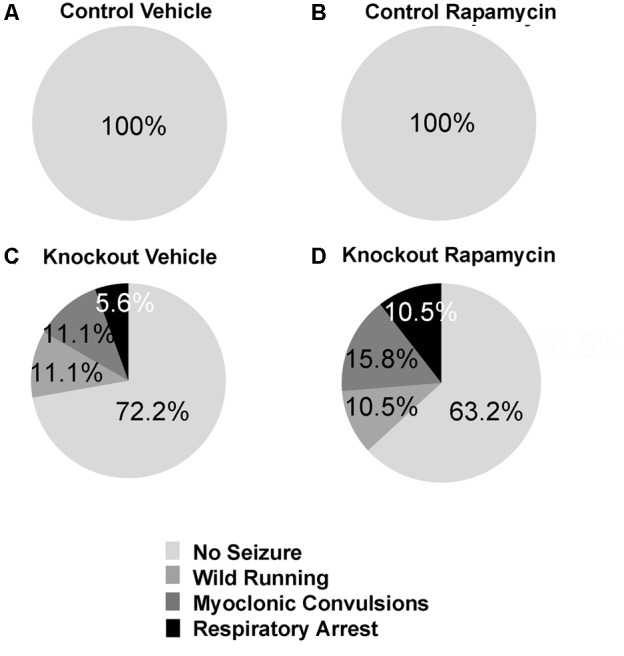
Rapamycin did not rescue audiogenic seizure susceptibility in *Fmr1* KO mice. **(A,B)** Neither control mice on vehicle (*n* = 17) or rapamycin treatment (*n* = 15) had any seizure activity. **(C)** *Fmr1* KO mice on vehicle treatment (*n* = 18) had a 27.8% seizure incidence. 11.1% were wild running, while 5.6% proceeded all the way to respiratory arrest. **(D)** *Fmr1* KO mice on rapamycin treatment (*n* = 19) had a 36.8% seizure incidence. 10.5% were wild running, while 10.5% proceeded all the way to respiratory arrest. This indicates that rapamycin was not effective in ameliorating the seizure phenotype in *Fmr1* KO mice.

We did not observe audiogenic seizures in either vehicle- or rapamycin-treated control mice (**Figures 7A,B**). Vehicle-treated *Fmr1* KO mice had a 28% seizure incidence (11% wild running, 11% myoclonic convulsions, and 6% respiratory arrest) (**Figure 7C**). Rapamycin-treated *Fmr1* KO had a 37% seizure incidence (11% wild running, 16% myoclonic convulsions, and 11% respiratory arrest) (**Figure 7D**). There were no statistically significant differences between vehicle and rapamycin-treated *Fmr1* KO mice.

## Discussion

The present study is, to our knowledge, the first study of the behavioral effects of chronic rapamycin treatment in *Fmr1* KO mice. We found that *Fmr1* KO mice have increased levels of p-S6 (a downstream target of mTORC1), and that rapamycin reverses this phenotype. However, rapamycin failed to reverse most of the behavioral phenotypes measured. Moreover, rapamycin exacerbated some of the abnormal behaviors resulting in further decreases in sleep duration and increased deficits in social interaction.

Increased activity of the mTORC1 pathway in the hippocampus of *Fmr1* KO mice has been reported previously (Sharma et al., 2010; Bhattacharya et al., 2012; Liu et al., 2012; Busquets-Garcia et al., 2013; Choi et al., 2016). Our present studies focused on the neocortex where we did not find an increase in p-mTOR or p-p70S6K, but we did find that p-S6, the downstream product of mTORC1, was increased at both phosphorylation sites (Ser235/236 and Ser240/244). Though rapamycin treatment reduced p-S6 levels, the lack of changes in p-mTOR and p-p70S6K suggest that rapamycin is reducing phosphorylation of S6 through a mechanism that is independent of mTORC1. This mechanism may be through mTORC2, which is supported by our results showing that rapamycin treatment reduced p-Akt at Ser473 in *Fmr1* KO mice. Whereas acute rapamycin is considered a selective mTORC1 inhibitor, chronic rapamycin treatment can also inhibit mTORC2 (Sarbassov et al., 2006). We posit that some of the effects of chronic rapamycin treatment on *Fmr1* KO mice may be due to mTORC2 inhibition.

In a recent paper that also examined signaling molecules in cortical lysates from young *Fmr1* KO mice (Sawicka et al., 2016), p-mTOR was not increased, but S6 phosphorylation at Ser235/236 was increased via an ERK-dependent kinase (p90S6K); S6 phosphorylation at Ser240/244 (mTOR/p70S6K-dependent) was unchanged. In adult *Fmr1* KO mice, this study further showed elevated ERK/p90S6K/S6 signaling in neocortex suggesting ERK/p90S6K (but not mTORC1) signaling dysregulation in neocortex. We did find that rapamycin reduced ERK, but we did not find any baseline genotype differences in p-ERK. It is possible that some of the differences between our present study and the results from Sawicka et al. (2016) could be due to the use of isoflurane anesthesia in the Sawicka et al. (2016) study; isoflurane has been shown to effect changes in translational control signaling pathways (Antila et al., 2017).

The effects of rapamycin treatment on behavior were negative and, in some cases, detrimental. Other studies have shown negative effects of rapamycin on behavior in control animals. In mice, acute rapamycin impaired performance on the Morris water maze (Ehninger et al., 2008), subacute rapamycin impaired performance on object place recognition (Zhou et al., 2013), and chronic rapamycin increased anxiety-like behavior (Russo et al., 2016) and resulted in deficits in social interaction (Reith et al., 2012). Similarly, studies in control rats have also demonstrated negative effects of rapamycin treatment. Acute rapamycin increased anxiety-like behavior (Hadamitzky et al., 2014), and chronic rapamycin treatment impaired performance on tests of learning and memory and increased anxiety-like behavior (Lu et al., 2015). In accord with these detrimental effects, it has been shown that long-term rapamycin treatment in young rats resulted in a decrease in progenitor cells in the dentate gyrus (Lu et al., 2015).

Whereas chronic rapamycin treatment has not been examined previously in *Fmr1* KO mice, there have been studies of the effects of acute treatment. In agreement with our results, acute rapamycin treatment in *Fmr1* KO mice also showed no effect on audiogenic seizure susceptibility (Osterweil et al., 2010). Moreover, in hippocampal slices, rapamycin treatment did not reverse the increased [^35^S]methionine/[^35^S]cysteine incorporation into protein (Osterweil et al., 2010). Another study (Price et al., 2007) addressed sensitization to pain. It was noted that acute rapamycin inhibited formalin- and DHPG-induced nociception in control animals but had no effect in *Fmr1* KO mice (Price et al., 2007), suggesting that the role of mTORC1 in nociception is altered in the absence of FMRP.

Numerous studies in *Fmr1* KO mice have addressed targets upstream of mTORC1. For example, targeting elements of the phosphoinositide 3-kinase (PI3K) pathway was efficacious in FXS models (Gross et al., 2015; Gantois et al., 2017; Monyak et al., 2017). Reducing the expression of PIKE rescued phenotypes in *Fmr1* KO mice, but PIKE reduction is thought, at least in part, to exert some of its effects in an mTOR-independent manner (Gross et al., 2015) Metformin, an AMPK activator, rescued FXS behavioral symptoms in both *Drosophila* and *Fmr1* KO mice (Gantois et al., 2017; Monyak et al., 2017). AMPK can act as an mTORC1 inhibitor (Xu et al., 2012), but AMPK has many other targets in addition to mTORC1 (Canto and Auwerx, 2010). Moreover, there are AMPK-independent effects of metformin suggesting additional mechanisms of action that have not been identified (Rena et al., 2013). Downstream of the mTORC1 pathway, genetic reduction of p70S6K1 reversed many deficits seen in *Fmr1* KO mice including elevated hippocampal protein synthesis, dendritic spine abnormalities, and deficits in learning and memory and social novelty interaction (Bhattacharya et al., 2012). Whereas these results suggested that targeting the mTORC1 pathway would be beneficial in FXS, results of our study in which we used pharmacological inhibition of mTORC1 do not support this idea.

It is possible that mTORC1 mediates too many effects to be a good drug target. Although clearly this is not the case in mouse models of TSC. TSC mice exhibit many similar behavioral phenotypes to *Fmr1* KO mice including social behavior abnormalities and learning and memory deficits, and these were rescued by chronic rapamycin treatment (Ehninger et al., 2008; Reith et al., 2012; Sato et al., 2012; Tsai et al., 2012). This suggests that although the mTORC1 pathway is implicated in both disorders with similar phenotypic outcomes, the molecular pathology underlying these disorders is quite different. Supporting this notion is the fact that a genetic cross between *Fmr1* KO and *Tsc2^+/-^* mice normalized long-term depression as well as performance in a contextual fear conditioning task (Auerbach et al., 2011).

Our results collectively indicate that rapamycin treatment is not efficacious, and in some respects, is detrimental in *Fmr1* KO mice. Other pathways should be explored for mechanism-based treatment strategies in *Fmr1* KO mice.

## Author Contributions

RS designed the study, performed the experiments, analyzed the data, interpreted the data, and drafted the manuscript. AS, IL, AC, and IM performed the experiments. AL analyzed the data. CS performed the experiments. CBS designed the study, interpreted the data, and drafted the manuscript. All authors have read and approved the final version of the manuscript.

## Conflict of Interest Statement

The authors declare that the research was conducted in the absence of any commercial or financial relationships that could be construed as a potential conflict of interest.

## Abbreviations

AMPK: adenosine monophosphate-activated protein kinase
ASD: autism spectrum disorders
FXS: Fragile X syndrome
KO: knockout
mTOR: mammalian target of rapamycin
PI3K: phosphoinositide-3 kinase
PIKE: PI3K enhancer
SEM: standard error of the mean
TSC: tuberous sclerosis complex
